# Effects of Major Components of Synovial Fluid on the Morphology and Wear Rate of Polyetheretherketone (PEEK) Particles under an Accelerated Wear Process

**DOI:** 10.3390/polym10060635

**Published:** 2018-06-08

**Authors:** Chen-Ying Su, Shih-Shuan Huang, Hsu-Wei Fang

**Affiliations:** 1 Department of Chemical Engineering and Biotechnology, National Taipei University of Technology, 1, Sec. 3, Zhongxiao E. Rd., Taipei 10608, Taiwan; chenying.su@ntut.edu.tw (C.-Y.S.); felaien@yahoo.com.tw (S.-S.H.); 2 Institute of Biomedical Engineering and Nanomedicine, National Health Research Institutes, No. 35, Keyan Road, Zhunan Town, Miaoli County 35053, Taiwan

**Keywords:** polymer, bio-tribology, joint prostheses

## Abstract

Wear particle-induced biological responses are the major factors for the failure of total joint arthroplasties, but it is possible to improve the lubrication and reduce the wear of an artificial joint system. Polyetheretherketone (PEEK), with ultra-high molecular weight polyethylene, is a suitable bearing material due to its resistance to fatigue strain. However, the effects of major compositions of synovial fluid on the wear of PEEK are unclear. We characterized the effects of three major components of synovial fluid including albumin, globulin, and phospholipids on the morphology and wear rate of PEEK wear particles. Our results demonstrated that the concentrations of albumin and globulin could affect the morphology of PEEK wear particles. In addition, a higher concentration of globulin and phospholipids (12.5 mg/mL) resulted in an increase in the amount of wear particles by 2.8- and 1.7-fold, respectively. In contrast, increasing albumin caused a reduction of wear particle numbers. These results indicate increasing concentration of albumin or reducing concentration of globulin or phospholipids has a better effect on reducing the numbers of wear particles and provides a potential solution of reducing PEEK wear particles, thus it can be more effectively applied in other biomedical systems.

## 1. Introduction

The performance of total joint arthroplasties (TJAs) is mainly determined by their tribological properties because the wear particles that are generated from the tribological process play a key role in the lifetime of artificial joint system [1,2,3]. In addition, wear particles may further induce biological responses from surrounding tissues and cells [4,5,6,7]. The main material for artificial joints is an ultra-high molecular weight polyethylene (UHMWPE), which has been proven to function well and is widely used clinically. However, it has been shown that the wear of UHMWPE and wear particles induced osteolysis and bone resorption [8,9,10]. The average life time of UHMWPE is around 10 years, and patients may have to undergo surgery again for joint replacement. Therefore, researchers have been looking for better bearing materials for TJA. Another polymeric material, polyetheretherketone (PEEK), has been introduced as bearing materials for TJAs due to its resistance to fatigue strain, and has been widely used for spinal instrumentation [11,12]. Therefore, PEEK has been considered as an alternative material for TJA device.

Many studies have shown that the size of wear particles is critical for particle-induced biological responses [13,14,15,16,17,18]. It has been demonstrated in vitro that UHMWPE wear particles with a mean size of 0.24 to 7.2 μm are more proinflammatory [13,14,16,17], while PEEK wear particles only induce cytotoxicity at a size larger than 10 μm [19]. Many factors can affect the sizes of wear particles, including the materials of contact surfaces and sliding conditions [20,21]. Human synovial fluid is mainly composed with albumin, globulin, lipid, and hyaluronic acid (HA), and these components may also contribute to determining the sizes of generated wear particles. Our previous study [22] has set up an accelerated wear testing protocol by sliding PEEK with microfabricated surfaces in a linear motion to generate large amounts of PEEK wear particles. Although the motion of artificial joints is rotational and linear, only the linear motion was investigated in this study.

We investigated PEEK wear characteristics under the lubrication of three major components of synovial fluid in this study. A combined analysis of the wear particle morphology and numbers of PEEK wear particles during an accelerated wear process could help us to understand the lubricating effects of synovial fluid on the characteristics of wear particles. Our results may provide feasibility for developing lubricating additives to reduce the PEEK wear particle-induced biological responses in TJAs.

## 2. Material and Methods

### 2.1. Materials

PEEK cylinder pins were obtained from A-SPINE Asia Co. Ltd. (Taiwan, Taibei) (batch number was SSR 0151). The pins were 6.35 mm in diameter and 25.4 mm in length with diamond turning on both end surfaces without polishing. Human serum albumin (HSA, Sigma-Aldrich A9511), (St. Louis, MO, USA) human immunoglobulin G (IgG, Sigma-Aldrich I4506), (St. Louis, MO, USA) and dipalmitoyl phosphatidyl choline (DPPC, Sigma-Aldrich P1652) (St. Louis, MO, USA) powders tested in this study were prepared in phosphate buffered saline (PBS, UniRegion UR-PBS001), (New Taipei City, Taiwan) with four different concentrations (0.1 mg/mL, 0.5 mg/mL, 2.5 mg/mL, and 12.5 mg/mL). Because PEEK is hydroscopic, all PEEK pins were presoaked in PBS for at least 30 days at 25 °C so they would not absorb HSA, IgG, or DPPC during the tests. Thus, the consistency of tests could be maintained.

### 2.2. Wear Process

**Figure 1:**
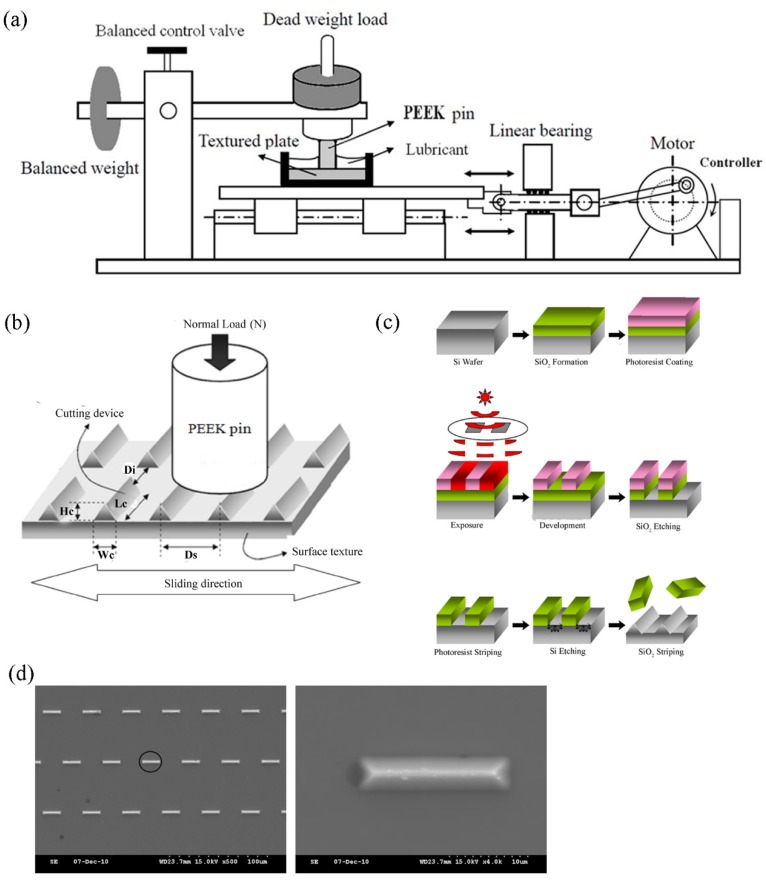
SEM images of the microfabricated surfaces. (**a**) Schematic of the setup for linear reciprocating wear testing. (**b**) Schematic of the particle generation in which microfabricated surface texture is rubbed against PEEK pin. Di: distance between adjacent features. Ds: pitch in the sliding direction. Lc: cutting-edge length. Wc: cutting-edge width. Hc: cutting-edge height. (**c**) Illustration of microfabricated surface textures process. (**d**) The wedges used in this study are 5 μm in width and 20 μm in length. The texture in the black circle is magnified on the right.

By rubbing PEEK pin with the cutting edges of the silicon surface textures, the PEEK wear particles were generated. ASTM F732 was used as a guideline. The setup of the system was described and shown in Figure 1a [23]. Before testing, the PEEK pin was weighed three times and the average was obtained. Linear reciprocating wear tests were performed at 25 °C under a nominal contact pressure of 1.5 MPa, a stroke length of 19 mm, and an average sliding speed of 57.2 mm/s in 5 mL of each lubricant for 6 hours (Figure 1b). After wear process, the PEEK pin was weighed three times and the average was obtained. Then the averaged weight of PEEK pin before the wear process was subtracted by the averaged weight after the process to obtain the weight loss. Wear rate was obtained when the weight loss (mg) of PEEK pin was divided by 360 min. Four pins were tested for each lubricant.

### 2.3. Microfabricated Surface Textures

The silicon wafer surface with controlled asperities was prepared by photolithography patterning and etching of the bulk substrate as previously described [24] and shown in Figure 1c. Once the surface textures were made, a layer of a 5-nm Chromium was coated onto the surface. In this study, the dimensions of the surface textures were measured from scanning electron microscopy (SEM) observations and the features were 5 μm in width and 20 μm in length (Figure 1d).

### 2.4. Isolation of the Wear Particles

PEEK particles were collected by repeated water rinsing of the sample in a sample holder, and parts that came into contact with particles were moved into a sterilized beaker. The process of isolation was previously described in detail [22].

### 2.5. Analysis of the Particles

After wear particles were isolated, they were examined by using a scanning electron microscope (SEM). Micrographs of the particles were then analyzed by using an image analyzer software (Scion Image, a personal computer version of NIH Image) to measure their dimensions. Measurements were made for at least 300 particles in each condition.

## 3. Results and Discussion

### 3.1. The Morphology of Wear Particles Depends on the Concentration of HSA and IgG but Not DPPC

**Figure 2:**
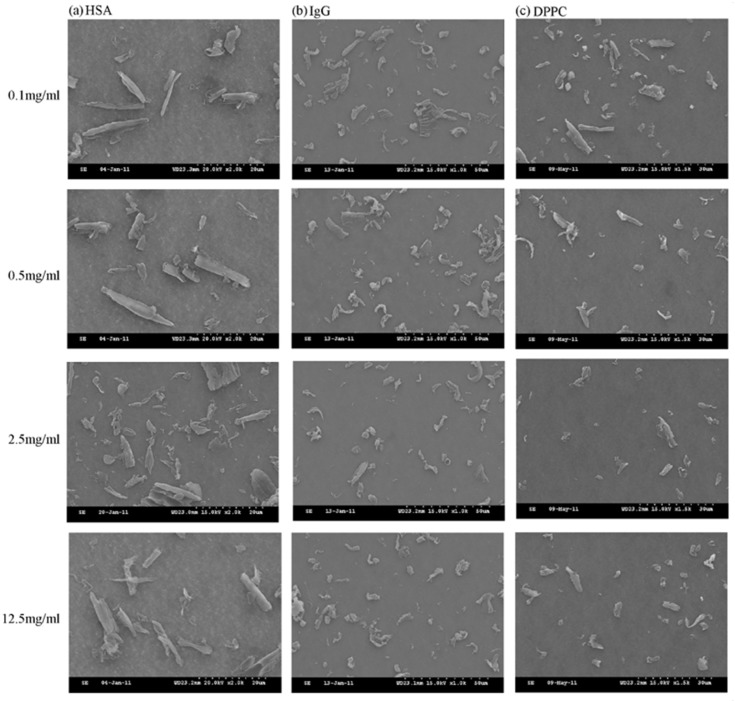
Morphology of PEEK wear particles under the lubrication of HSA, IgG or DPPC solution. (**a**) SEM images of the PEEK wear particles caused by rubbing PEEK pin against microfabricated surfaces in 0.1, 0.5, 2.5, and 12.5 mg/mL HSA solution (2000× magnification). (**b**) SEM images of the PEEK wear particles caused by rubbing PEEK pin against microfabricated surfaces in 0.1, 0.5, 2.5, and 12.5 mg/mL IgG solution (1000× magnification). (**c**) SEM images of the PEEK wear particles caused by rubbing PEEK pin against microfabricated surfaces in 0.1, 0.5, 2.5, and 12.5 mg/mL DPPC solution (1500× magnification).

**Figure 3:**
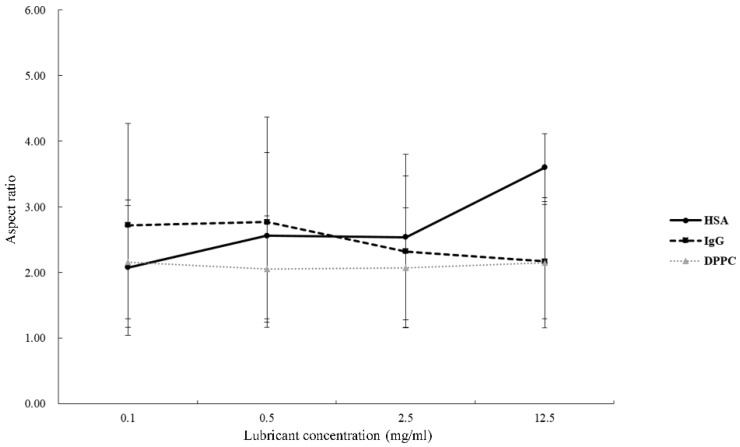
Aspect ratio of PEEK wear particles under the lubrication of HSA, IgG or DPPC solution. Plots of aspect ratio and the wear rate in different concentrations of HSA, IgG or DPPC solution.

Table 1 listed the length, width, aspect ratio of the PEEK wear particles, as well as mass wear and wear rate under the lubrication of HSA, IgG, and DPPC, respectively. The SEM images of the wear particles under the lubrication of HSA, IgG, and DPPC were shown in Figure 2. Aspect ratio was obtained by dividing the length into the width of wear particles, and an increase in aspect ratio was associated with rod-like shape while the decrease in aspect ratio indicated granular shape. When PEEK pin was articulated in HSA solution, increasing the concentration of HSA resulted in an increase in the length and aspect ratio (Figure 2a and Figure 3). When PEEK pin was rubbed in IgG solution, both particle length and width were increased but aspect ratio was decreased suggesting that increased IgG concentration was associated with generating granular shape wear particle (Figure 2b and Figure 3). The morphology of wear particles was not changed dramatically when PEEK pin was rubbed in DPPC solution, suggesting that the sizes of wear particles were not affected by the concentration of DPPC (Figure 2c).

Our results demonstrated that increasing concentration of DPPC had no effect on the aspect ratio of PEEK wear particles. The changes in aspect ratio of particles were opposite when rubbing PEEK in HSA and IgG: a higher concentration of HSA resulted in a rod-like shape of particles while a higher concentration of IgG caused a granular shape of particles (Figure 2a,b). It has been shown that when polymers were absorbed in water, various effects could occur including a reduction in strength, a decrease in modulus of elasticity, an increase in the elongation to break, or swelling of the surface layers resulting in differential expansion [25,26].

**Figure 4:**
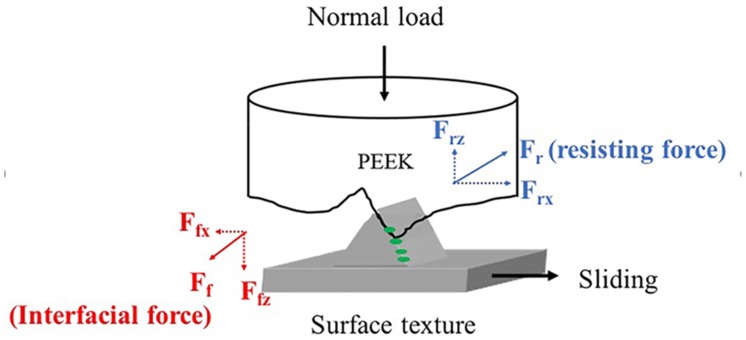
Mechanical analysis of the resisting force and interfacial friction for the surface feature sliding over PEEK process. F_rz_ is upward direction of the resisting force; F_rx_ is horizontal direction of the resisting force; F_fz_ means downward direction of the interfacial friction; F_fx_ is horizontal direction of the interfacial friction. Green circles represent the testing components such as HSA, IgG, or DPPC.

The wear process of the surface feature over PEEK results in a resisting force F_r_ on PEEK (Figure 4). The vertical direction of this resisting force F_rz_ pushes up the PEEK while lateral movement continues. The interfacial friction (F_f_) is manifested as an encounter force to prevent the PEEK being pushed away. If the component causes an increase of interfacial friction, it will lead to a larger downward force F_fz_. Therefore, the surface feature shall slide longer to complete a wear process and results in an increase in aspect ratio of particles. In contrast, the accumulated layer of the component at the interface may reduce the actual penetration depth of the surface feature into PEEK. Thus, it would further shorten the lateral sliding distance, resulting in a reduction of aspect ratio of particles. Therefore, it is possible that IgG formed a thicker layer at the interface and reduced the penetration depth feature into PEEK. PEEK pin then decreased its lateral sliding distance, and was easily broken to form a granular shape of particles when concentration of IgG was increased. In contrast, immersing PEEK in HSA may increase the downward force, resulting in an increase of wear particles. However, the results suggested that DPPC may not change the penetration depth of the surface feature into PEEK, thus the aspect ratio remained the same when increasing the concentration of DPPC.

Both rod-like and granular shapes of carbon fiber reinforced-PEEK (CFR-PEEK) wear particles were identified in macrophages in human tissue retrieval studies, although both cases were not associated with joint arthroplasty failure [27,28]. In patients going through knee revision surgery, a small amount of synovial tissue was collected and varies sizes of CFR-PEEK particles formed conglomerates near to the vessel [29]. None of the biological responses were reported, and although PEEK wear particles were found in those patients, it is difficult to draw a conclusion of whether the size and the shape of PEEK wear particles influence biological response in humans. The testing procedure we applied here provided a rapid method for PEEK wear particle generation under different concentrations of HSA, IgG, and DPPC, thus providing a rapid way to compare morphology of PEEK wear particles. Furthermore, the wear process should be conducted by joint simulators in the future to imitate the clinical situation.

### 3.2. Wear Rates Depend on the Concentration of HSA, IgG, and DPPC

**Figure 5:**
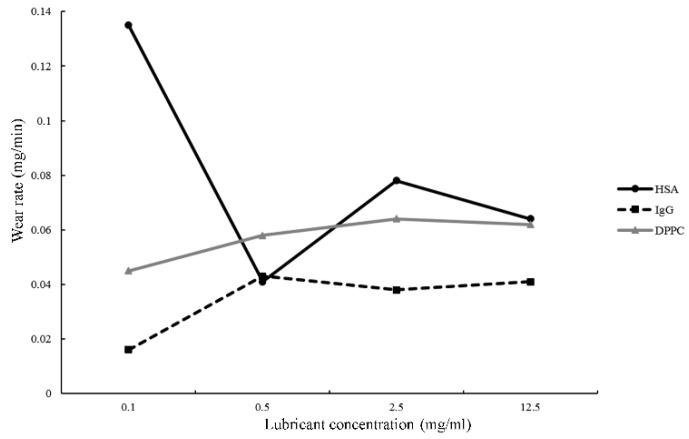
Wear rate of PEEK wear particles under the lubrication of HSA, IgG, or DPPC solution. Plots of aspect ratio and the wear rate in different concentrations of HSA, IgG or DPPC solution.

When PEEK pin was rubbed in HSA solution, increasing HSA concentration resulted in reducing wear rate (Figure 5 and Table 1). Although the reduction was more obvious when articulation of PEEK pins was in 0.5 mg/mL of HSA than in a higher concentration of HSA, the trend of mass wear generation was overall decreased, resulting in an increase of HSA. In contrast, increasing the concentration of both IgG and DPPC resulted in an increase of wear rate (Figure 5 and Table 1).

Our results demonstrated that the total wear rate was the lowest when PEEK was rubbing in IgG. The friction between artificial joints is boundary lubrication according to the Stribeck curve, and the lubricant will accumulate and form a thin layer on the surface to protect the joints [30]. It is possible that IgG forms a thicker layer on the surface of PEEK and the microfabricated edges resulting in reducing the penetration depth of the cutting edge into PEEK and reducing the numbers of wear particles. However, increasing the concentration of IgG still resulted in an increase of wear rate suggesting that lower concentration of IgG might be more suitable for protecting the surface of PEEK. In contrast, HSA may form a much thinner layer, thus higher concentration of HSA is needed to reduce the penetration of the cutting edge into PEEK. Therefore, a higher concentration of HSA caused a decrease of wear particles.

DPPC carries positively charged quaternary ammonium ion, and usually could adhere to negatively charged proteoglycans on the surface of cartilage [31]. The strong adhesion between DPPC and cartilage can reduce friction, and higher concentrations of DPPC can increase its lubricating ability [32]. In addition, DPPC can form DPPC bilayers and it has been shown that a reduction in wear between joint and cartilage can occur as long as DPPC bilayers are not separated from the surface [33]. However, PEEK is not cartilage thus there might not be strong adhesion between PEEK and DPPC. Increasing the concentration of DPPC may result in stronger adhesion among DPPC bilayers, and result in adhesive wear in our system, thus higher wear rate was obtained. Taken together, our results demonstrated that lower concentration of IgG and DPPC can be good lubricants in terms of reducing PEEK wear rate. In addition, the cost of manufacturing PEEK is high. Our results also provided additional information about the factors that could affect PEEK wear rate, thus the potential lubricants can be applied in reducing PEEK wear particles in biomedical systems for prolonging the lifetime of PEEK.

## 4. Conclusions

An accelerated wear testing procedure was established successfully to evaluate the lubrication effects of albumin, globulin, and phospholipids in this study. The morphologies of PEEK wear particles have been characterized, and the effects of different lubricants on the aspect ratio of wear particles have been investigated. The results for the wear rate can help us to discuss the lubricating ability of these three components. Although there is no conclusive trend for the wear rate obtained in this study, our scale-up testing system for generating PEEK wear particles has given us a rapid screening method for identifying the role of lubricants in the boundary lubrication of artificial joints. This testing method may be beneficial for further developing lubricating additives for total joint arthroplasties in the future.

## Figures and Tables

**Table 1:** Morphology and wear of PEEK particles in different components of synovial fluid.

Component	Concentration (mg/mL)	Particle length (μm)	Particle width (μm)	Aspect ratio	Mass wear (mg)	Wear rate (mg/min)
HSA	0.1	4.33 ± 3.31	2.14 ± 1.51	2.08 ± 1.03	48.8	0.136
	0.5	3.47 ± 2.63	1.41 ± 0.85	2.56 ± 1.27	14.7	0.041
	2.5	4.90 ± 3.70	1.96 ± 1.23	2.54 ± 1.26	28.0	0.078
	12.5	10.52 ± 7.01	2.96 ± 1.08	3.60 ± 2.16	22.9	0.064
IgG	0.1	5.77 ± 3.17	2.12 ± 0.97	2.72 ± 1.55	5.8	0.016
	0.5	6.11 ± 3.24	2.39 ± 1.07	2.77 ± 1.60	15.6	0.043
	2.5	4.91 ± 4.07	2.08 ± 1.28	2.32 ± 1.15	13.8	0.038
	12.5	7.75 ± 3.55	3.80 ± 1.54	2.17 ± 0.87	14.7	0.041
DPPC	0.1	4.21 ± 2.65	1.97 ± 1.01	2.16 ± 0.86	16.3	0.045
	0.5	4.28 ± 2.81	2.19 ± 1.32	2.05 ± 0.81	20.9	0.058
	2.5	4.88 ± 3.34	2.42 ± 1.52	2.07 ± 0.91	23.1	0.064
	12.5	4.64 ± 3.04	2.20 ± 1.22	2.15 ± 0.99	22.2	0.062
